# Apigenin Induces Autophagy and Apoptosis in Chemoresistant Glioblastoma Cells and Inhibits Tumorigenicity Associated with Regulation of Immunomodulatory Proteins and Glial Cells Response

**DOI:** 10.3390/cells14191552

**Published:** 2025-10-03

**Authors:** Paulo Lucas Cerqueira Coelho, Cleonice Creusa dos Santos, Alessandra Bispo da Silva, Karina Costa da Silva, Monique Reis de Santana, Balbino Lino dos Santos, Giselle Pinto de Faria Lopes, Marie Pierre Junier, Hervé Chneiweiss, Vivaldo Moura-Neto, Maria de Fátima Dias Costa, Suzana Braga-de-Souza, Silvia Lima Costa

**Affiliations:** 1 Laboratory of Neurochemistry and Cell Biology, Department of Biochemistry and Biophysics, Institute of Health Sciences, Federal University of Bahia, Salvador 40110–100, BA, Brazil; paulocoelhoba@gmail.com (P.L.C.C.); cleonicemev@gmail.com (C.C.d.S.); alebispo18@hotmail.com (A.B.d.S.); enf.karinac@gmail.com (K.C.d.S.); moniquereisant@gmail.com (M.R.d.S.); balbino.lino@univasf.edu.br (B.L.d.S.); fatima@ufba.br (M.d.F.D.C.); 2 College of Nursing, Federal University of Vale do São Francisco, Av. José de Sá Maniçoba, S/N–Centro, Petrolina 56304–917, PE, Brazil; 3 Division of Bioproducts, Department of Marine Biotechnology, Instituto de Estudos do Mar Paulo Moreira (IEAPM), Arraial do Cabo 28930–000, RJ, Brazil; giselle.faria@gmail.com; 4 Center for Neuroscience at Sorbonne University (NeuroSU), Institute of Biology Paris Seine (IBPS), CNRS, INSERM, Sorbonne University, 75005 Paris, France; marie-pierre.junier@inserm.fr (M.P.J.); herve.chneiweiss@inserm.fr (H.C.); 5 State Institute of the Brain Paulo Niemeyer, Rio de Janeiro 20230–024, RJ, Brazil; vivaldomouraneto@gmail.com; 6 National Institute for Translational Neuroscience/National Council for Scientific and Technological Development (INCT/CNPq), Neurociência Translacional (INNT), Carlos Chagas Filho 373, Rio de Janeiro 21941–902, RJ, Brazil; 7 Neurociência Translacional (INNT), Institute of Biomedical Sciences/Federal University of Rio de Janeiro (ICB/UFRJ), Carlos Chagas Filho 373, Rio de Janeiro 21941–902, RJ, Brazil

**Keywords:** glioblastoma stem cells, flavonoid, resistance, apoptosis, immunomodulation

## Abstract

Background: Glioblastomas (GBMs) are the most aggressive and common neoplasms that affect glial cells, presenting rapid growth, invasion, and resistance to treatments. Studies have demonstrated the potentially inhibitory effect of flavonoids on glioblastoma cells’ stemness and viability. However, further research is needed to explore sensitivity and the mechanism of action in chemoresistant cells. Methods: In this study, we characterized the impact of apigenin treatment on the viability and differentiation of human GBM cells in vitro and its effects on tumorigenesis and regulation of the inflammatory response in vivo. Results: The flavonoid apigenin reduced the viability of U-251 cells, patient-derived cells TG-1 and OB-1 stem cells in a dose-dependent manner, associated with the induction of acidic vesicle organelles formation and apoptosis. Treatment with apigenin also inhibited migration and induced neural differentiation in the remaining viable cells, characterized by a decrease in the expression of the precursor marker nestin and an increase in the expression of astrocyte and neuron markers, GFAP and β-III tubulin, respectively. The xenotransplantation of apigenin-pretreated U251 cells into rat brains did not lead to tumor formation, unlike untreated cells. The surrounding area of transplanted untreated U251 cells exhibited reactive microglia and astrocytes, along with increased VEGF expression, which was absent in implant sites of apigenin-pretreated GBM cells. Moreover, in this implant area, we observed a significant decrease in the expression of mRNA for inflammatory factors IL-1β, TNF, and NOS2, and the downregulation of IL-10 and IL-4. Conclusions: These results demonstrate that apigenin inhibits the growth of tumoral cells, affecting the viability of tumor stem cells and impairing tumorigenicity, while altering the regulatory profile of immunomodulatory proteins. Therefore, this flavonoid can be considered for further studies to determine its use as an adjuvant to the treatment of human GBMs.

## 1. Introduction

Gliomas are malignant intracranial tumors that affect glial cells in the Central Nervous System (CNS). Their classification according to the World Health Organization (WHO) varies in four degrees, with human glioblastoma (GBM) being the most aggressive [1]. The incidence of GBM has increased in recent decades, showing that it is the most common type of primary brain neoplasm, affecting individuals across all ages. However, its prevalence is higher in white men and people between 45 and 70 years of age [2].

The GBM morphological aspects, including high density of vascularity, intense cellular and endothelial proliferation, and rapid growth and invasiveness, contribute to its high recurrence rates. The cellular constitution in a single tumor presents significant variation, which, in turn, can hinder the therapeutic protocol for GBMs [3]. A population of chemotherapy-resistant tumor stem cells, responsible for glioma recurrence, is found within the tumor mass [4,5,6].

Currently, the treatment protocol is multisystemic, combining surgical removal, chemotherapy, and radiotherapy [7]. Among the therapeutic agents used, temozolomide (TMZ) is noteworthy. Despite advances in clinical oncology, the prognosis for patients remains poor, with an average life expectancy of 15 months [8]. The failure of GBM therapy is mainly related to chemoresistance in a population of stem cells [7,8]. In this context, research has explored alternative therapeutic strategies to improve glioma treatment efficacy, including the use of natural compounds.

Flavonoids are plant-derived polyphenolic compounds known for their biological properties, particularly their antitumor effects, which have attracted significant scientific interest [9,10]. The flavonoid apigenin (4′,5,7-trihydroxyflavone) has been extensively investigated in biological research, primarily for its anti-inflammatory and antioxidant properties, as well as its antitumor effects [11,12]. In glioma cells, apigenin exhibited cytotoxicity, acting as a potent inducer of cell cycle arrest and apoptosis, and was capable of inducing microglia/macrophage response [13,14]. However, further extensive research is needed to explore the sensitivity and the mechanism of action in chemoresistant cells, and the efficacy of this compound for adjuvant treatments.

This study characterized the impact of apigenin treatment on the viability and differentiation of U-251, TG-1 and OB-1 human GBM stem cells in vitro. Additionally, we characterized in vivo the effects of apigenin on the tumorigenicity of GBM cells following xenotransplantation into the brains of immunocompetent rats.

## 2. Materials and Methods

### 2.1. Cell Culture

The U-251 human GBM cell line (09063001 Sigma, St. Louis, MO, USA) was cultured to confluence in polystyrene plates (TPP, Trasadingen, Switzerland) using Dulbecco’s modified Eagle’s medium (DMEM; Cultilab, Campinas, Brazil) with 100 IU/mL penicillin (Gibco^®^, Grand Island, NY, USA), 100 mg/mL streptomycin (Gibco^®^, Grand Island, NY, USA), 7 mmol/L glucose (Sigma, Saint Louis, MO, USA), 2 mmol/L L-glutamine (Sigma, Saint Louis, MO, USA), 0.011 g/L pyruvic acid (Sigma, Saint Louis, MO, USA), and 10% fetal calf serum (FCS), (Gibco^®^, Grand Island, NY, USA) as described previously [15]. The TG-1 and OB-1 (TG1C1) glioblastoma stem-like cells were obtained from human tumors [4,16] and maintained in DMEM/F12 medium supplemented with 1 mM L-glutamine, 25 mM glucose, 10 mM HEPES (Sigma, Saint Louis, MO, USA, and growth factors N2, G5, and B27 (Invitrogen, Thermo Fisher Scientific, Waltham, MA, USA), as described previously by Assad-Khan et al. [16]. All cultures were kept in a humid atmosphere of 95% air and 5% CO_2_ at 37 °C.

### 2.2. Drugs and Treatment

Apigenin (5,7,4′-trihydroxyflavone) was extracted from the leaves of *Croton betulaster* Müll., a shrub belonging to the Euphorbiaceae family [17], in Chapada Diamantina, Bahia, Brazil. Aerial parts of the plant were collected and identified, and a voucher specimen was deposited in the herbarium of the Federal University of Bahia (ALCB number 031762), Brazil. The extraction was performed using 600 g of air-dried leaves, first with hexane (Sigma-Aldrich, Saint Louis, MO, USA), then with dichloromethane (Sigma-Aldrich, Saint Louis, MO, USA), and finally with methanol (Sigma-Aldrich, Saint Louis, MO, USA). Column chromatography of the dichloromethane extract (45 g, Sigma-Aldrich, Saint Louis, MO, USA) with increasing ethyl acetate (EtOAc, Sigma-Aldrich, Saint Louis, MO, USA) in hexane yielded 81 milligrams of apigenin, identified through nuclear magnetic resonance (NMR) analysis. The apigenin extract (>97% purity) was dissolved in dimethyl sulfoxide (DMSO; Sigma, St. Louis, MO, USA) at a concentration of 100 mM and stored at −20 °C in the dark. For the toxicity assay, cells were exposed to apigenin at concentrations ranging from 1 to 100 μM or maintained in a control condition (0.1% DMSO). Based on prior cytotoxicity results in glioblastoma cell lines, a concentration of 50 μM apigenin and 0.05% DMSO was used for all subsequent assays. The choice of apigenin concentration was based on dose-response tests in previous studies in vitro that observed a selective cytotoxic concentration for glioma cells and the potential to modulate microglia immune response [14] and based on anti-glioma effective and selective concentrations of other polyhydroxylated flavonoids, such as quercetin and naringenin [9,15,18].

### 2.3. Cytotoxicity Assay

The cytotoxicity of apigenin on U-251 cells was evaluated using the MTT assay (3-(4,5-dimethylthiazol-2-yl)-2,5-diphenyltetrazolium bromide) (Invitrogen, Thermo Fisher Scientific, Waltham, MA, USA). U-251 cells were cultured in 96-well plates (TPP) at a density of 2 × 10^5^ cells/cm^2^ and exposed to apigenin at concentrations of 1, 10, 50, and 100 μM, or to a control (0.1% DMSO) for 48 h. Two hours before the exposure ended, the culture medium was replaced with MTT solution (5 mg/mL in DMEM) and incubated at 37 °C with 5% (*v*/*v*) CO_2_. Afterward, a lysis buffer containing 20% sodium dodecyl sulfate (SDS) (Sigma-Aldrich, Saint Louis, MO, USA), 50% acetic acid (Sigma-Aldrich, Saint Louis, MO, USA), and 2.5% HCl (Sigma-Aldrich, Saint Louis, MO, USA) was added, and the plates were left overnight for the formazan crystals to dissolve. Optical density was measured at 540 nm using a Bio-Rad 550 PLUS Spectrophotometer (Bio-Rad, Santo Amaro, Brazil).

TG-1 and OB-1 cells were plated in 96-well plates at a density of 2 × 10^4^ cells per well and treated with apigenin at concentrations of 1, 10, 50, and 100 μM, along with a control of 0.1% DMSO. They were incubated at 37 °C with 5% CO_2_ for 48 h. Cell viability was assessed using the WST-1 assay (4-[3-4-iodo-phenyl)-2-(4-nitrophenyl)-2H-5-tetrazolio]-1,3-benzene disulfonate, Roche, Île-de-France, France), where 10% WST-1 was added after incubation, followed by 3 h of incubation. Absorbance was measured at 430 nm using a microplate reader (Expert Plus V1.4, ASYS, Salzburg, Austria). Three independent experiments were performed, each with eight replicates per condition.

### 2.4. Differentiation and Morphological Characterization

Morphological changes were assessed using phase-contrast microscopy on control and treated U-251 cells, seeded in 40 mm polystyrene dishes at a density of 2 × 10^5^ cells/cm^2^. An optical phase microscope (Nikon TS-100, Nikon, Melville, NY, USA) with a digital camera (Nikon E-4300, Nikon, Melville, NY, USA) was used for analysis. Immunocytochemical staining for nestin (a marker of immature nerve cells), glial fibrillary acidic protein (GFAP, a marker of astrocytes), and β-III tubulin (a neuronal marker) was performed to examine morphological changes and differentiation. U-251 cultures were rinsed with phosphate-buffered saline (PBS) and fixed in cold methanol at −20 °C for 10 min. TG-1 and OB-1 cells were harvested, washed, and then smeared onto SuperFrost slides, followed by fixation in cold methanol for 20 min. After fixation, cells were rewashed and incubated with 0.3% PBS-Triton X-100 (Hexis científica, Jundiai, SP, Brazil) and 5% bovine serum albumin (BSA) (Sigma-Aldrich, Saint Louis, MO, USA) for 30 min. Nonspecific antibody binding was blocked by pre-incubating the plates with 3% bovine serum albumin (BSA) in PBS. Cells were incubated with mouse monoclonal primary antibody against nestin (clone 3B4, 1:500; Santa Cruz, Dallas, TX, USA), rabbit polyclonal antibody against GFAP (1:500; Dako, Glostrup, Denmark), or mouse monoclonal anti-β-III tubulin antibody (1:500; Sigma, St. Louis, MO, USA). Cells were diluted in PBS with 1% BSA and incubated for 12 h at 4 °C with slow agitation. After washing three times with PBS, they were incubated with secondary antibodies: Alexa Fluor 488-conjugated goat anti-rabbit IgG (1:400, A11008, Molecular Probes, Eugene, OR, USA), Alexa Fluor 488-conjugated goat anti-mouse IgG (1:400, A11001, Molecular Probes, Eugene, OR, USA), or Alexa Fluor 555-conjugated goat anti-rabbit IgG (1:400, A21434, Molecular Probes, Eugene, OR, USA). Control cells were treated without primary antibodies. Nuclear chromatin was stained with 5 μg/mL of 4′6-diamidino-2-phenylindole (DAPI, Molecular Probes, Eugene, OR, USA) for 10 min at room temperature in the dark. Cells were then analyzed using an Olympus BX-2 epifluorescence microscope (Olympus Corporation, Tokyo, Japan), capturing images from ten randomized fields per condition. All assays were conducted at least three times.

### 2.5. Cell Adherence Assay

U-251 cells were cultured and plated in 96-well plates (BD Biosciences, BD BioCoat Poly-D-Lysine, Franklin Lakes, NJ, USA) at a density of 5 × 10^3^ cells per well. Adherent cells were counted 24 h post-incubation and expressed as a percentage of the total. Images were captured and analyzed using a Nikon TS-100 optical phase microscope with a Nikon E-4300 digital camera (Nikon Corporation, Tokyo, Japan).

### 2.6. Neural Lineage Differentiation

To induce astrocytic differentiation, TG-1 and OB-1 cells were treated with 5% serum. For neuronal differentiation, the cells were plated on poly-ornithine- and laminin-coated plates (Thermo Fisher Scientific, Waltham, MA, USA). After 10 days, the medium was replaced with neurobasal supplemented with N2, B27, and FGF2 (Invitrogen, Thermo Fisher Scientific, Waltham, MA, USA), Four days later, FGF2 was removed, and then the medium was switched to neurobasal with B27, CNTF, and BDNF (Miltenyi Biotech, Bergisch Gladbach, NW, Germany). All supplements were for stem cells.

### 2.7. Apoptosis Evaluation

Apoptosis was evaluated by monitoring phosphatidylserine externalization using an annexin V-FITC/propidium iodide (PI) staining kit (BD Biosciences Clontech, Mountain View, CA, USA). U-251 cells were cultured in 40-mm plates and treated with apigenin (50 μM) or control (DMSO 0.1%) for 48 h. Approximately 1.5 × 10^4^ cells were resuspended in 100 µL of binding buffer containing annexin V-FITC (0.125 µg/mL) and PI (5 µg/mL), then incubated for 15 min at room temperature in the dark. Flow cytometry analysis was performed using a BD FACSort machine (BD Biosciences, San Diego, CA, USA), measuring annexin V fluorescence in the FL1 channel and PI fluorescence in the FL2 channel. At least 10,000 events were recorded, with annexin V-positive (apoptotic) and PI-positive (necrotic) cells reported as percentages of the total cells counted. Experiments were repeated at least three times.

Caspase-3 immunofluorescence staining of U-251 cells was performed to determine the apoptotic pathway. The cells were cultured in 96-well plates (TPP) at a density of 2 × 10^5^ cells/cm^2^ and exposed to apigenin (50 μM), or a control (0.1% DMSO). After 48 h of treatment cells were rinsed with PBS and fixed in cold methanol at −20 °C for 10 min. After fixation, cells were rewashed and incubated with 0.3% PBS-Triton X-100 for 15 min, then incubated with 5% BSA for 1 h. Cells were incubated with rabbit anti-active-caspase-3 antibodies (1:400; #9661 Cell signaling technology, Inc., Danvers, MA, USA) at 4 °C overnight. After washing three times with PBS, they were incubated with a secondary antibody (Alexa Fluor 594-conjugated goat anti-rabbit IgG (1:1000, A 11012, Invitrogen/Thermo Fisher). Control cells were treated without primary antibodies. Nuclear chromatin was stained with 5 μg/mL of 4′6-diamidino-2-phenylindole (DAPI, Molecular Probes, Eugene, OR, USA) for 10 min at room temperature in the dark. Cells were then analyzed using an Olympus BX-2 epifluorescence microscope, capturing images from ten randomized fields per condition. All assays were conducted at least three times.

### 2.8. Acidic Vesicular Organelles

The ability of apigenin to induce the formation of acidic vesicular organelles (AVOs) was confirmed using acridine orange staining, followed by flow cytometry and fluorescence microscopy. Cells were treated with apigenin (50 μM) or a control (0.1% DMSO) for 48 h, then incubated with acridine orange dye (1 μg/mL) (Sigma, Saint Louis, MO, USA) for 15 min at room temperature. After washing with PBS and treating with 0.25% trypsin, cells were centrifuged and resuspended in 250 μL of PBS with 2% BSA for flow cytometry analysis. Using BD FACSort (BD Biosciences), at least 10,000 events were recorded. FL1-positive cells were considered alive, whereas FL3-positive cells were identified as autophagic. Results were expressed as the percentage of autophagic cells relative to the total cell count. At least three independent experiments were conducted, and fluorescence microscopy (DCF7000, Leica, Wetzlar, Germany) was used to capture 10 images per treatment for further analysis.

### 2.9. Migration Assay

U-251 cells were seeded in 24-well plates at a density of 5 × 10^4^ cells per well. After forming a confluent monolayer, a uniform wound was created using a 200 μL pipette tip, and the cultures were rinsed with PBS to remove detached cells. They were treated with apigenin (50 μM) or a control (0.1% DMSO), with or without 10% FBS, and incubated at 37 °C and 5% CO_2_. Cultures were observed using phase-contrast microscopy, and images were taken at 0-, 24-, 48-, and 72-h post-treatment. Wound area quantification was conducted with ImageJ 1.33u (NIH, Bethesda, MD, USA).

### 2.10. Tumorigenesis Analysis In Vivo

Three-month-old male Wistar rats, weighing 300–350 g, were divided into two groups of six. One group received unilateral implants of U-251 human glioblastoma cells in 0.1% DMSO for 24 h, while the other group received implants of U-251 glioblastoma cells treated with apigenin (50 μM) for the same duration. The study was approved by the Ethics Committee at the Federal University of Bahia (registration number 0272012). The rats were anesthetized with ketamine (100 mg/kg, Sigma-Aldrich, Saint Louis, MO, USA) and xylazine (25 mg/kg, Sigma-Aldrich, Saint Louis, MO, USA) and secured in a stereotactic apparatus (Stoelting™, Stoelting LLC, Wood Dale, IL, USA). A small hole was drilled in the skull to inject tumor cells into the caudate putamen. U-251 cells, either in control conditions or treated with apigenin, were detached from culture plates using 0.25% trypsin (Sigma-Aldrich, Saint Louis, MO, USA) and resuspended in DMEM. Viable cells were counted using trypan blue staining. A total of 500,000 cells in a 5-μL volume were injected using a Hamilton syringe. After surgery, the animals were kept in individual cages and monitored daily. Thirty days after tumor cell injection, the rats were anesthetized and transcardially perfused with 4% paraformaldehyde (PFA, 158127, Sigma-Aldrich, St. Louis, MA, USA) in phosphate-buffered saline (PBS) for perfusion fixation. Brains were dissected, postfixed in cold 4% PFA for 24 h, and stored at −4 °C before processing.

### 2.11. Hematoxylin–Eosin Staining and Tumor Volume Calculation

Brain tissues were dehydrated using increasing concentrations of ethanol and xylol (Sigma-Aldrich, Saint Louis, MO, USA), then embedded in paraffin. Tissue sections were cut at 4 μm on glass slides. After deparaffinization and hydration, the slides were stained with hematoxylin (Merck, Darmstad, HE, Germany) for 5 min, rinsed with tap water, dipped in acidic alcohol, and washed again. After, they were counterstained with eosin solution (Merck, Darmstad, HE, Germany) for 30 s and rinsed with distilled water. Serial sections of 40 μm were prepared from each brain and collected in a 24-well multiwell plate. Each slice was placed consecutively in a well until the entire brain was sectioned. For brain tumor volume calculation, slices from one tube were transferred to a new well and incubated for 20 min in 0.3% PBS-Triton X-100 with DAPI (1:4000; Sigma-Aldrich). The tumor area of each slice was captured using fluorescence microscopy (Leica) and processed with ImageJ 1.33u (NIH, Bethesda, MD, USA) to determine the tumor areas. The formula used for tumor volume calculation was tumor volume = slice size (40 μm) × number of slices (24) × sum of tumor areas from one well. Tumor volumes were calculated by analyzing slices from at least two wells.

### 2.12. Immunohistochemical Assay

To perform the immunohistochemical reactions, brains were prefixed and immersed in a 30% sucrose solution for 3 days. Slices of 25 μm were cut using a cryostat (SLEE MAIZ Cryostat MCT, SLEE medical GmbH, Schwalbach am Taunus, Germany) at –20 °C and mounted on Superfrost glass slides (Sigma, St. Louis, MA, USA). The tissue sections were washed three times with PBS and then incubated in a 0.3% PBS–Triton X-100 solution for 20 min, followed by incubation in 5% NGS (Thermo Fisher Scientific, Austin, TX, USA) for 1 h to block non-specific binding. They were incubated with primary specific antibodies for anti-GFAP (1:200, Z0334, DAKO, Glostrup, Denmark), anti-Iba-1 (1:100, WAKO, 019-19741, Saitama, Japan) from rabbit, and anti-VEGF (1:200, C-1 7269, Santa Cruz Biotechnology Dallas, TX, USA) from mouse, overnight at 4 °C. After washing three times in PBS, secondary antibodies (Alexa Fluor 488-conjugated goat anti-rabbit IgG and goat anti-mouse IgG, both 1:400 (Boehringer, Mannheim, Germany) were added for 2 h. The nuclei were stained with DAPI (5 μg/mL, Molecular Probes, Eugene, OR, USA) for 10 min, followed by PBS washing and mounting with Fluoromount (Sigma-Aldrich, St. Louis, MA, USA). Finally, the slices were analyzed using fluorescence microscopy (Leica, Wetzlar, Germany), with 10 images captured per condition.

### 2.13. RNA Isolation and cDNA Synthesis

RNA was isolated from the rat brain area xenotransplanted with U-251 cells under control conditions (0.05% DMSO) or treated with 50 μM apigenin for 24 h, along with the contralateral area without implants. Samples of 1 mm^3^ were taken from both areas of three animals in each group. RNA was extracted using the Trizol^®^ reagent protocol (Catalog # 15596026, Invitrogen Life Technologies, Carlsbad, CA, USA), according to the manufacturer’s specifications. The concentration and purity of total RNA were determined using a Nanospectrum (K23-0002, Kasvi, São José dos Pinhais, PR, Brazil). The Ambion^®^ DNA-freeTM kit (Thermo Fisher Scientific, Austin, TX, USA) was used to treat the RNA (2.5 μg) with DNase (Ambion cat# AM1906, Thermo Fisher Scientific, Austin, TX, USA) for contaminant removal. The cDNA was synthesized by Superscript^®^ VILO™ Master Mix (Life Technologies, Invitrogen, cat# MAN0004286, Carlsbad, CA, USA) following the manufacturer’s instructions.

### 2.14. qPCR

The TaqMan Gene Expression Assay (Applied Biosystems, Foster City, CA, USA) was used to quantify the mRNA expression of genes encoding proteins of interest by quantitative PCR (qPCR). It was performed using two primers to amplify the sequence of interest and the specific Taqman^®^ MGB probe (Applied Biosystems, Carlsbad, CA, USA) with FAM fluorophore, along with the TaqMan^®^ Universal Master Mix II and UNG 82 (Catalog # 4,440,038 Invitrogen, Life Technologies™, Carlsbad, CA, USA). The assay identifications for the genes quantified in this study were: TNF (Rn01525859_g1), IL1B (Rn00580432_m1), IL10 (Rn00563409_m1), IL4 (Rn01456866_m1), NOS2 (Rn00561646_m1), and IGF1 (Rn00710306_m1) from rats. Real-time PCR was performed by the QuantStudioTM 7 Flex Real-Time PCR System (Applied Biosystems, USA). Thermocycling conditions were performed according to the manufacturer’s instructions. β-actin (Rn00667869_m1 for rat-derived samples and Hs99999903_m1 for human-derived samples) and HPRT1 (Rn01527840_m1 for rat, and Hs02800695_m1 for human) were used as endogenous controls for normalization. Data analysis considered the 2^−ΔΔCT^ method [19]. Results were obtained from at least three independent experiments.

### 2.15. Statistical Analysis

Results are presented as mean ± SEM and as a percentage of the control (DMSO = 100%). A one-way ANOVA, followed by the Student–Newman–Keuls test, identified significant differences among groups with one varying parameter. A Student’s *t*-test with Welch’s correction was used for comparison between the two groups. A *p*-value of <0.05 was considered significant, and all analyses involved three independent experiments.

## 3. Results

### 3.1. Apigenin Inhibits the Viability of Human Glioblastoma Cells

**Figure 1:**
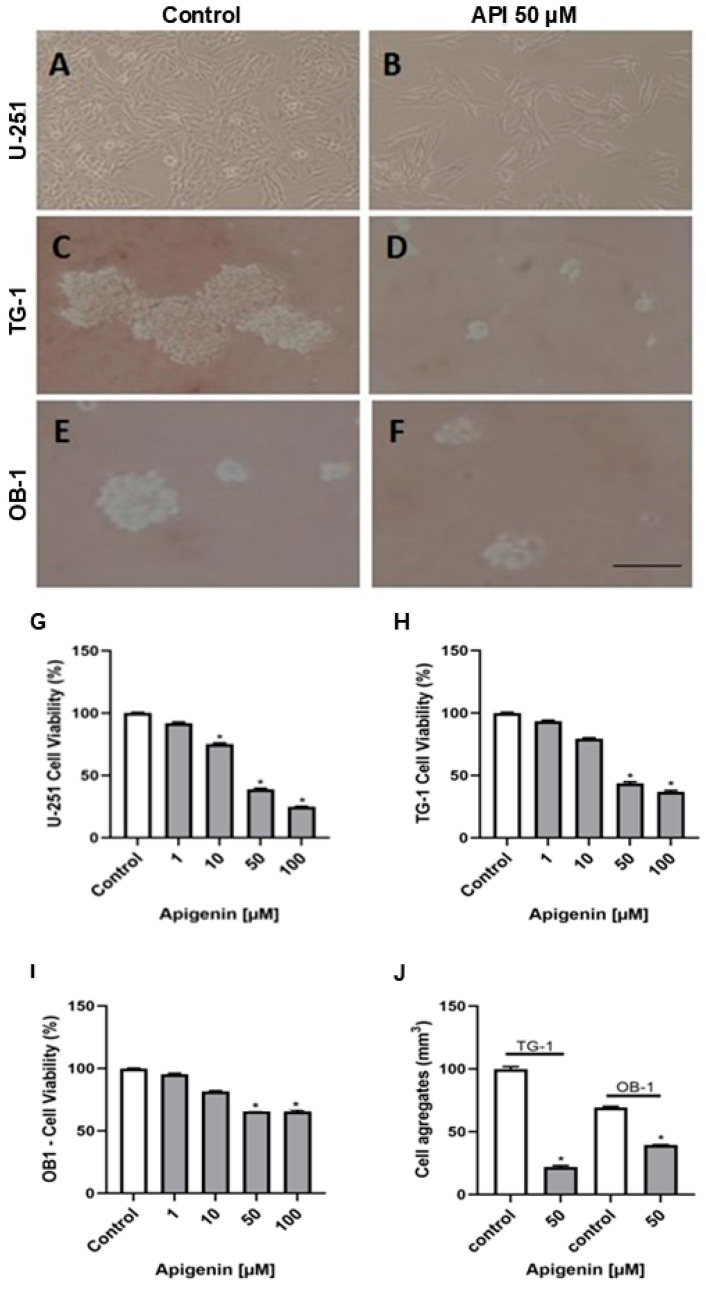
Apigenin inhibits the cell viability of human glioblastoma cells. Contrast phase micrography of U-251 (**A**,**B**), TG-1 (**C**,**D**), and OB-1 (**E**,**F**) cells, treated with 0.1% DMSO (control) or 50 µM apigenin (API) for 48 h. Percentage of cell viability of U-251 (**G**), TG-1 (**H**), or OB-1 (**I**) cells treated with 1, 10, 50, and 100 µM apigenin (API) for 48 h compared to control (0.1% DMSO). Viability assay performed by MTT for U-251 cells or WST1 for TG-1 and OB-1 cells. * *p* < 0.05 by one-way ANOVA followed by the Newman-Keuls post-test. (**J**) Cell aggregate volume (mm^3^) of TG-1 and OB-1 cells, 48 h after treatment with 50 µM apigenin, compared to the control. The volume was calculated as the total aggregate of cells, using an unpaired *t*-test with Welch’s correction for unequal variances. * *p* < 0.05. Results are represented as the mean ± standard deviation of three independent experiments, expressed as a percentage of the control, which is considered 100%. Scale bar 40 µm.

The cytotoxic effects of apigenin (1–100 μM) on U-251 glioblastoma cells were assessed by using the MTT assay. A significant reduction in viable U-251 cells (*p* < 0.05) was observed after 48 h of exposure to 10 μM apigenin. Notably, the most pronounced effects were detected at concentrations of 50 and 100 μM under the same experimental conditions (Figure 1G). TG-1 and OB-1 cell viability significantly decreased (*p* < 0.05) at 50 and 100 μM apigenin after 48 h of treatment (Figure 1H,I). The effects of 50 μM apigenin in U-251 cells, TG-1 and OB-1 stem cells were analyzed using phase-contrast microscopy after 48 h of exposure. The control U-251 cells exhibited high cellular density with cell size variation depending on the culture density, as observed by phase-contrast microscopy (Figure 1A). Treatment with apigenin (50 μM) reduced the monolayer integrity and cell density of U-251 cells (Figure 1B). Glioma stem cells TG-1 and OB-1 formed cell aggregates in control conditions, which, due to their quiescent state, tended to proliferate and form colonies. The number of TG-1 aggregates was larger than that of OB-1, suggesting a higher proliferation rate (Figure 1C,J). When these cells were treated with apigenin (50 µM), there was a breakdown of cell colonies, indicating an interruption of the self-renewal capacity of these stem cells. A significant decrease in cellular aggregates was observed in both cultures after treatment (Figure 1D,F,J). Another approach to assessing the toxicity of substances to glioma stem cells is measuring the size of spheroid cell aggregates after treatment. Exposure to apigenin (50 μM) for 48 h reduced the volume of cell aggregates of TG-1 and OB-1 glioblastoma stem cells (Figure 1J). Among the two cell lines, TG-1 cells exhibited more sensitivity to apigenin across all assays.

### 3.2. Apigenin Induces Differentiation in Glioblastoma Cells

**Figure 2:**
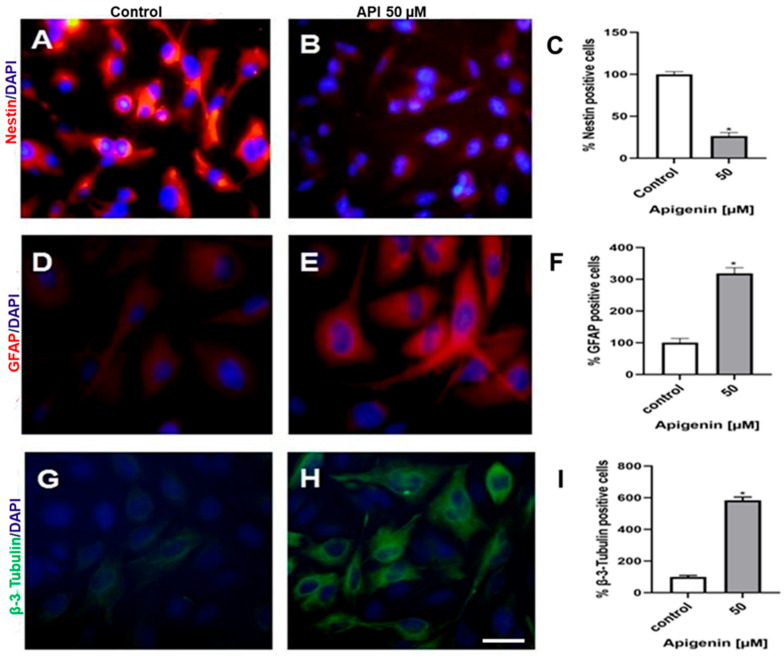
Apigenin induces differentiation in U-251 glioblastoma cells. Immunofluorescence of (**A**,**B**) the immature cell marker nestin (red), (**D**,**E**) the astrocyte marker glial fibrillary acidic protein (GFAP, red) and (**G**,**H**) the neuron marker β-3 tubulin (green) of U-251 cells in control conditions (0.1% DMSO) and treated with 50 μM apigenin (API) for 48 h. Quantification of (**C**) nestin, (**F**) GFAP, and (**I**) β-3 tubulin positive cells comparing the two different culture conditions. The nuclear chromatin was stained by DAPI (blue). Results represented the mean ± standard deviation of three independent experiments. The control (0.1% DMSO) was considered as 100%. * *p* < 0.05 by unpaired *t*-test. Scale bar: 50 μm.

The effects of apigenin were analyzed using phase microscopy and immunocytochemical staining for nestin (immature glial cells), GFAP (mature astrocytes), and β-3 tubulin (differentiated neurons). The control U-251 cells exhibited a bipolar, fibroblast-like phenotype, characterized by a uniformly high pattern of nestin expression. In contrast, GFAP and β-III tubulin expressions were low and heterogeneous, restricted to the perinuclear region of a subpopulation of cells (Figure 2A,D,G). After U-251 cells were treated with 50 μM apigenin for 48 h, a reduction in cellular density was observed. Treated cells exhibited an expansion of thin cellular processes, accompanied by higher GFAP and β-III tubulin expression, and very weak nestin expression, indicating a reduction in cell number and differentiation (Figure 2B,E,H).

### 3.3. Apigenin Accelerates Differentiation in Long-Term Glioma Stem Cell Cultures

**Figure 3:**
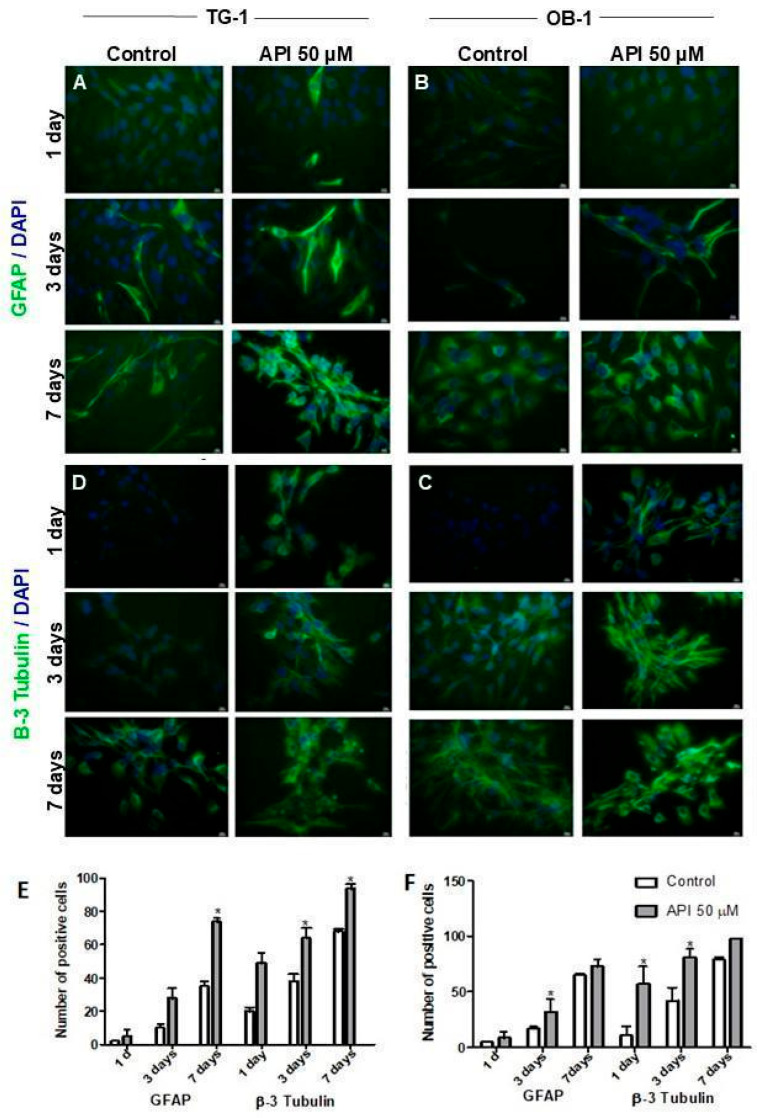
Apigenin promotes the differentiation of glioblastoma stem cells in long-term culture. TG-1 and OB-1 cells were treated with 50 μM apigenin (API) or 0.05% DMSO (control) for 1, 3, and 7 days. Immunoexpression of GFAP (green) in TG-1 (**A**) and OB-1 (**B**) cells. Immunoexpression of β-3 tubulin (green) in TG-1 (**C**) and OB-1 (**D**) cells. The nuclear chromatin was stained by DAPI (blue). Quantification of GFAP and β-3 tubulin positive cells in TG-1 (**E**) and OB-1 (**F**) cultures. Results represented the mean ± standard deviation of three independent experiments. * *p* < 0.05 by unpaired *t*-test. Scale bar: 50 μm.

To further evaluate whether apigenin influenced and/or differentiated the state of the glioma stem cell along the astroglial and neuronal lineages, TG-1 and OB-1 cells were exposed to apigenin (50 µM) for 1, 3, and 7 days. Exposure to apigenin increased the number of cells expressing differentiation markers characteristic of astrocytic (GFAP) and neuronal (β-III tubulin) profiles (Figure 3A–F). The expression of both markers increased over time, particularly in TG-1 cultures.

### 3.4. Apigenin Promoted Adhesion and Differentiation of U-251 Induced Glioblastoma Stem Cells

**Figure 4:**
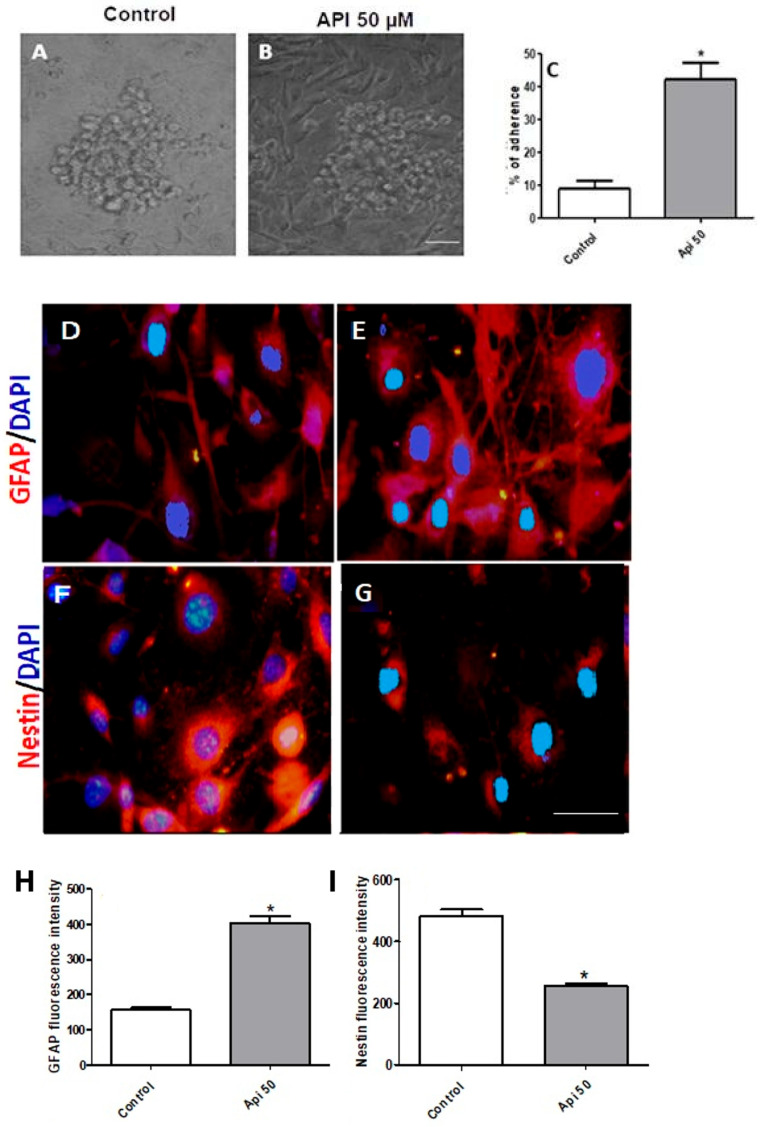
Apigenin promoted cell adhesion and differentiation in U-251 induced glioblastoma stem cells. U-251 cells were previously cultured with a stem cell conditioned medium for 7 days, and the resulting aggregates were treated with (**A**) 0.1% DMSO (control) or with (**B**) 50 µM apigenin (API) for 48 h, as illustrated by contrast phase micrography. (**C**) The percentage of adherent cells was analyzed using Adobe Photoshop. Immunoexpression (red) of GFAP (**D**,**E**) and nestin (**F**,**G**) in the two different conditions. The nuclear chromatin was stained by DAPI (blue). Quantification of (**H**) GFAP, and (**I**) nestin fluorescence intensity, comparing the two different conditions. Results are represented as the mean ± standard deviation of three independent experiments, expressed as a percentage of the control (0.05% DMSO), which is considered 100%. * *p* < 0.05 by unpaired *t*-test. Scale bar: 100 μm.

To investigate whether apigenin could promote tumoral adhesion of induced stem-like cells, U-251 was cultured in glioma stem cells conditioned medium (Figure 4A). After 7 days, U-251 induced stem cell colonies were treated with 50 µM apigenin for 48 h. This treatment enhanced cell adhesion on permissive substrates, increased membrane extensions, and reduced sphere formation (Figure 4B,C). Additionally, apigenin increased the expression of neuronal differentiation marker GFAP (Figure 4E,H) while decreasing the expression of the undifferentiated marker nestin (Figure 4G,I).

### 3.5. Apigenin Induces Apoptosis and Acidic Vesicular Organelles (AVOS) Formation in Human Glioblastoma Cells

**Figure 5:**
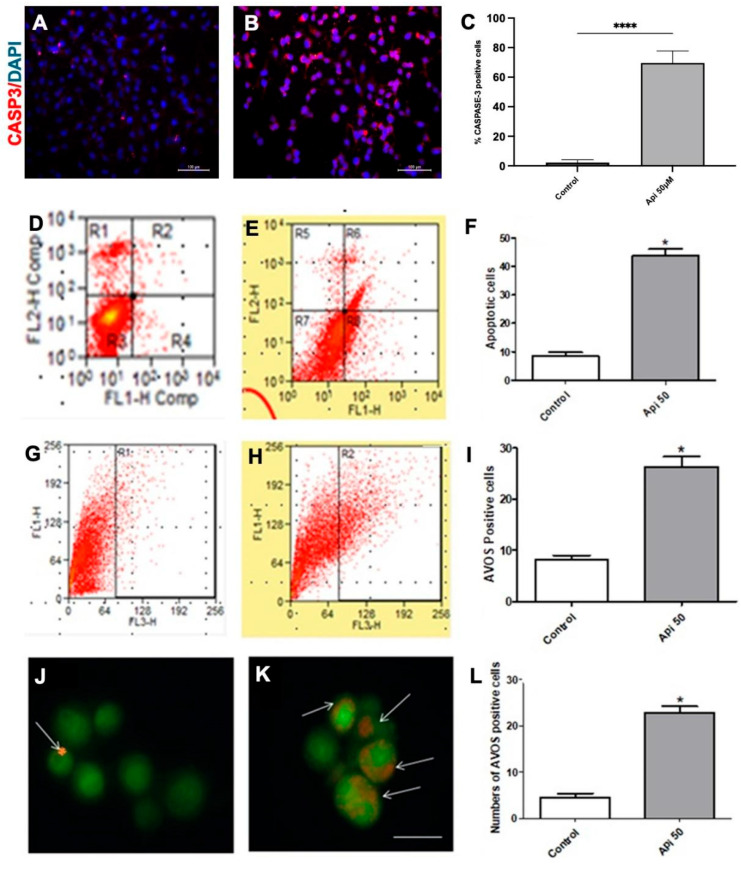
Apigenin induces apoptosis and the formation of acidic vesicular organelles in U-251 human glioblastoma cells. (**A**) Immunofluorescence for caspase-3 (red) in U-251 cells treated with 0.1% DMSO (control) or (**B**) 50 µM apigenin for 48 h. (**C**) Quantification of caspase-3 positive cells. The nuclear chromatin was stained by DAPI (blue). Annexin-V/propidium iodide flow cytometry of U-251 cells treated with (**D**) 0.1% DMSO (control) or (**E**) 50 µM apigenin (API) for 48 h. (**F**) Quantification of apoptotic cells analyzed by Annexin-V/Propidium iodide flow cytometry assay. Acridine orange flow cytometry in U-251 cells treated with (**G**) 0.1% DMSO (control) or (**H**) 50 µM apigenin (API) for 48 h. (**I**) Quantification of acidic vesicular organelles (AVOs) positive cells was quantified by acridine orange staining using a flow cytometry assay. (**J**) Fluorescence microscopy showing autophagic vacuoles stained by acridine orange (arrows) of U-251 cells treated with 0.1% DMSO (control) or (**K**) 50 µM apigenin for 48 h. Scale bar: 100 μm. (**L**). Quantification of AVOs positive U-251 cells by acridine orange fluorescence. Results represented the mean ± standard deviation of three independent experiments. * *p* < 0.05 and **** *p* < 0.0001 by unpaired *t*-test. A two-way ANOVA followed by the Student–Newman–Keuls post-hoc test was used. Flow cytometry fluorescence reading performed on the FACS Calibur (Becton & Dickinson) apparatus (**D**,**E**) for Annexin V on FL-1 channel and propidium iodide (PI) on FL-2 channel, respectively; (**F**) for acridine orange on FL-3 channel.

The study investigated the effects of apigenin (50 µM) on inducing apoptosis and necrosis in glioma cells using annexin V/propidium iodide staining, followed by flow cytometry, and immunofluorescence assay for caspase-3. Apigenin induced apoptosis in these conditions was also characterized by increased caspase-3—positive U251 cells from 2.1 ± 1.9% in control to 69.7 ± 8.2% in treated cells (Figure 5A–D). After 48 h of treatment, U-251 cells exhibited a significant rate of apoptosis, with 43.45 ± 5.19% of cells testing positive for annexin V (see Figure 5E,F). In contrast, the control group (treated with 0.1% DMSO) showed only 6.58 ± 1.33% annexin V-positive cells (see Figure 5D,F). In addition to apoptosis, apigenin (50 µM) was found to promote autophagy, as demonstrated through acridine orange staining, flow cytometry, and immunofluorescence analysis. The treated cells displayed a notable autophagy rate, with 25.14 ± 8.43% of cells testing positive for acridine orange, compared to just 6.53 ± 0.48% in the control conditions (0.1% DMSO) (see Figure 5G–I). Furthermore, fluorescence microscopy confirmed these results, showing a significant increase in acridine orange staining following treatment with 50 µM apigenin. The percentage of acidic vesicular organelles (AVOs) within the cells increased to 23.12 ± 2.15% following apigenin treatment, compared to 4.72 ± 1.29% in the control group (0.1% DMSO) (Figure 5J–L). AVOs are indicative of autophagy and occur earlier than apoptosis. These results suggest that apigenin treatment leads to a substantial increase in autophagic vacuole formation, followed by a subsequent induction of apoptosis.

### 3.6. Apigenin Inhibits U-251 Cell Migration

**Figure 6:**
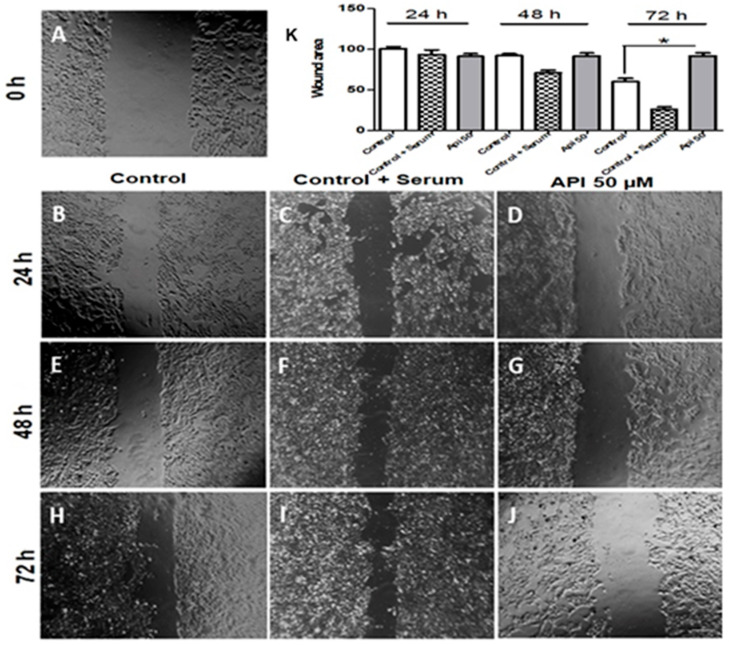
Apigenin inhibits U-251 cell migration. U-251 human glioblastoma cells were cultured with 0.05% DMSO (without fetal bovine serum) (control), or with 0.05% DMSO with 10% serum (control + serum), or with 50 μM apigenin (API 50 μM). (**A**) Contrast phase microscopy of the initial wounded area at 0 h. The migration of cells after (**B**–**D**) 24 h, (**E**–**G**) 48 h, and (**H**–**J**) 72 h. (**K**) Percentage of the wound area over time. Results are represented as the mean ± standard deviation of three independent experiments, with the control after 24 h set at 100% of the wound area. * *p* < 0.05 by one-way ANOVA followed by the Newman-Keuls post-test compared to control. Scale bar: 20 μm.

To evaluate the potential of apigenin to inhibit cell migration, a monolayer scratch assay was performed. U-251 glioma cells exhibited high migratory capacity in control cultures (0.05% DMSO) without FBS and with 10% FBS (positive control), resulting in almost 100% confluence at the end of 72 h (Figure 6I). This effect was particularly more evident when compared to the initial wounded area at 0 h (Figure 6A). Cell migration was reduced by 50 μM apigenin at 24, 48, and 72 h compared to controls. The mechanical injury of the U-251 cell monolayer showed decreased cellularity with apigenin compared to the control (Figure 6D,G,J). Notably, at 24 h, the wound area in apigenin-treated cells remained similar to that of the control (Figure 6K).

### 3.7. Apigenin Inhibited the Growth of U-251 Tumor Xenograft

**Figure 7:**
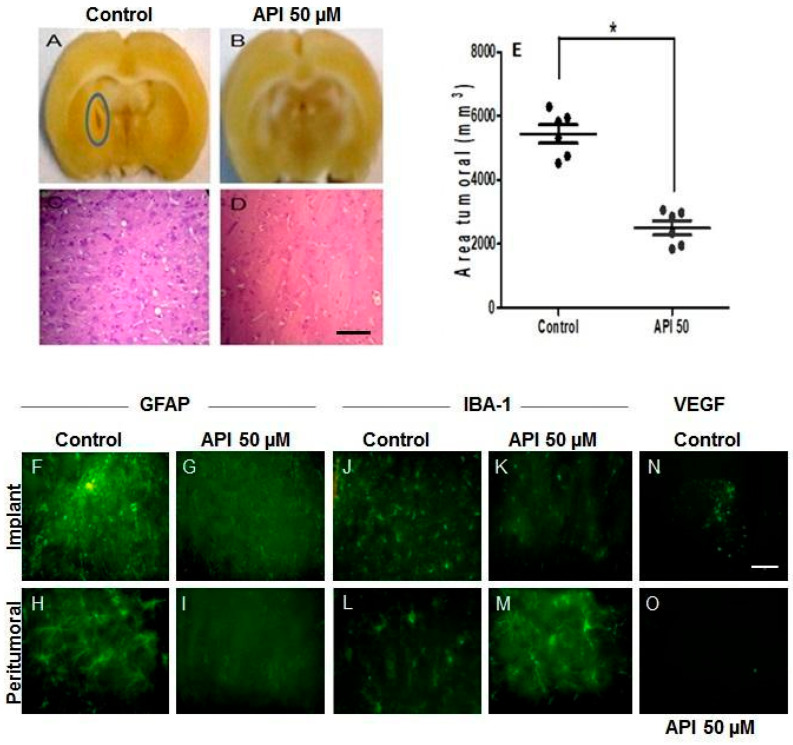
Apigenin inhibited the growth of U-251 tumor xenograft. (**A**,**B**) Macroscopic evaluation of brain rats 30 days after intracranial injection of 5 × 10^5^ U-251 cells pretreated with 0.05% DMSO (control) or 50 µM apigenin (API) for 24 h. Blue circle in A: tumor necrosis. (**C**,**D**) Hematoxylin/eosin staining of brain coronal sections from rats sacrificed 30 days after intracranial injection of U-251 cells in the two different conditions. Scale bar: 2 mm. (**E**) Graphic showing the volume of U-251 xenografts in rat brain with intracranial injection of U-251 cells, comparing the two conditions (* *p* < 0.05). (**F**–**M**) Immunostaining for GFAP and IBA-1 in the implant and peritumoral areas of control and apigenin-treated animals. (**N**,**O**) VEGF expression in the implant area of control and apigenin-treated animals. Scale bar = 100 μm.

The antitumorigenic effect of apigenin (50 µM) in vivo was evaluated to better simulate tumor microenvironment interactions, providing a deeper understanding of the phenomena underlying tumor pathogenesis. Wistar rats’ brains were studied for tumor formation following 30 days of xenotransplantation of U-251 human glioblastoma cells, after a 24 h pre-treatment with apigenin (50 μM) in vitro and untreated. The xenotransplantation was done in only one hemisphere of each brain. The contralateral hemisphere was not injected with U-251 glioblastoma cells. In the brains of control animals that received xenotransplants of U-251 cells pretreated with 0.05% DMSO, macroscopic lesions with darker central areas—indicative of necrosis—were observed in the xenotransplanted region (Figure 7A). In contrast, in the brains of animals that received U-251 cells pretreated with 50 μM apigenin, predominantly whitish and homogeneous areas were noted in the xenotransplantation region (Figure 7B). Histological analyses further revealed that in the control animals, the xenografted tumor infiltrated brain parenchyma, forming a solid tumor mass (Figure 7C). This tumor mass exhibited all the microscopic histopathological features necessary for a diagnosis of glioblastoma (GBM) according to WHO classification, including cellular atypia, the presence of mitotic figures, endothelial vascular proliferation, and necrosis. The animals that received the U-251 cells pretreated with apigenin 50 μM showed a reduced number of tumoral cells and increased tumor necrosis (Figure 7D). Additionally, tumor volume decreased (Figure 7E). To observe the behavior of glial cells in the presence of U-251 human glioblastoma cells, we investigated the morphology and activation of astrocytes and microglia through immunohistochemistry for GFAP and Iba-1. In the brain hemispheres of animals that received glioblastoma cells under control conditions, we observed a higher number of GFAP-positive, reactive astrocytes with well-defined cellular processes (Figure 7F). These GFAP-positive astrocytes were evenly distributed in the peritumoral area.

In contrast, in the brain hemispheres of animals injected with cells pretreated with apigenin, the proportion of GFAP-positive astrocytes in the tumor implant areas was reduced, displaying a less reactive phenotype compared to the control group (Figure 7G). The analysis of microglial morphology and activation, performed using Iba-1 immunohistochemistry, revealed an increased proportion of microglia in the tumor implant region under control conditions. There was a predominance of microglia exhibiting branched morphology (Figure 7J). This branched morphology was also observed in the peritumoral area; however, the number of activated microglia in the peritumoral area was lower than in the tumor implant area (Figure 7L). In animals injected with cells that had been previously treated with apigenin, both the implanted area and the peritumoral area showed a similar reduction in the proportion of microglia, with a predominance of amoeboid-shaped microglia, which is characteristic of activated microglia (Figure 7K–M). Moreover, we verified the vascular endothelial growth factor (VEGF) expression to assess neovascularization. We observed a reduction in VEGF expression in the tumor area of the brains from animals xenotransplanted with apigenin-treated cells, compared to the brain tissue of control animals that received cells not treated with apigenin (Figure 7N,O).

### 3.8. Xenotransplant of Apigenin-Treated GBM Cells Altered the Immune Profile in the Brain Tumor Area

**Figure 8:**
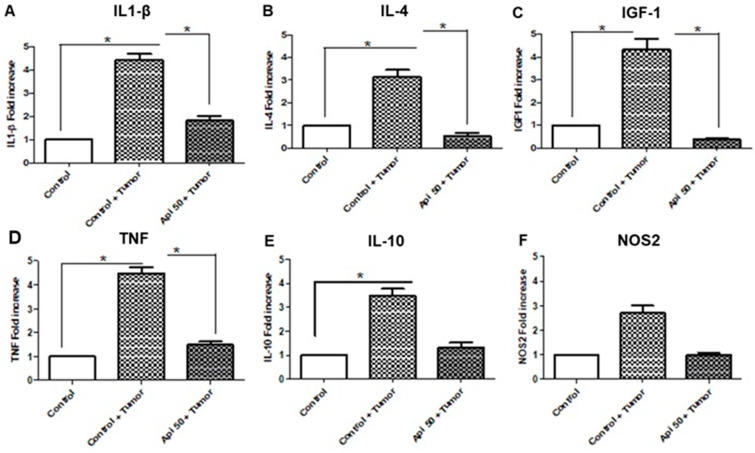
Apigenin altered the immune profile from a TH2 to a TH1 response. (**A–F**) Real-time PCR analysis of: (**A**) IL1-β, (**B**) IL-4, (**C**) IGF-1, (**D**) TNF, (**E**) IL-10, (**F**) NOS2 in rat brain without (control) or with intracranial injection of U-251 cells treated with 0.05% DMSO (control GBM cells) or 50 µM apigenin (API 50 + GBM cells). Results are represented as the mean ± standard deviation compared to the control without tumor, using one-way ANOVA and an unpaired *t*-test with Welch’s correction, * *p* < 0.05.

To assess apigenin’s potential to modulate inflammatory gene expression, RT-qPCR was performed using brain tissue with xenografts of U-251 cells to characterize the tumor microenvironment response profile. The reduced capacity of the immune response in the glioblastoma microenvironment is mainly due to the immunosuppressive response. Microglia and astrocytes are immunoreactive and secrete cytokines and trophic factors that influence tumor growth and migration. Brains of animals that received apigenin-GBM—pretreated cells showed a significant decrease in mRNA expression levels for IL-1β, IL-4, and TNF, when compared with GBM untreated cells (Figure 8). However, the TNF and IL1-β RNA values were greater than those of IL-4, suggesting an exacerbated anticancer inflammatory response in brains that received apigenin-GBM—pretreated cells. In addition, IL-10 and NOS2 expression levels were found to be increased in the GBM xenograft group compared with healthy tissue, consistent with the immunosuppressive and proinflammatory profile of the glioblastoma microenvironment. In the apigenin-treated group, a tendency toward downregulation of both IL-10 and NOS2 was observed, suggesting a potential modulatory effect of apigenin. Finally, to assess the prognosis of these animals, we verified the gene expression of IGF-1, a protein produced by the liver, which is elevated in the case of glioblastoma. A reduction in the levels of IGF-1 in the brain tissue of animals xenotransplanted with cells pretreated with apigenin was observed, compared to the expression levels in the brains of animals that received xenotransplantation of control tumor cells (Figure 8).

## 4. Discussion

In this study, the flavonoid apigenin exhibited a critical role in tumorigenesis profile, considering viability, growth, stemness, invasiveness, neovascularization and immunomodulatory effects on the U-251 glioblastoma cells, as well as on the TG-1 and OB-1 patient-derived glioblastoma stem-like cells. These findings align with previous research highlighting the concentration-dependent antitumor potential of flavonoids [11,12,13,14,15,18]. In addition, apigenin treatment inhibits the proliferation and migration of glioblastoma cells, which is consistent with previous studies in glioma cell lines [14,20,21,22]. Furthermore, the volume of cell aggregates was also reduced in glioblastoma stem cell cultures.

It was observed that the process of cell death resulting from apigenin treatment in vitro occurs to induce apoptosis through caspase-3 activation and acidic vesicular organelles formation, suggesting the phenomenon of autophagy may contribute to the induction of apoptosis and the death of tumor cells [23]. In this context, a drug with such effects may be effective as an antitumor agent also in the context of GBM targeting drug resistance [24,25]. Other studies have demonstrated that apigenin exerts anti-cancer effects via apoptosis and cell death in cancer cells [26]. In this study, the results suggest that apigenin may reduce U-251 cell proliferation by promoting both apoptosis and autophagy, two fundamental mechanisms associated with antitumor drugs. Caspase-3 is a key executioner enzyme in the apoptotic cascade, and its activation indicates progression towards programmed cell death [27]. An increase in the number of caspase-3–positive cells was observed, highlighting the potential of this flavonoid to induce apoptosis in GBM cells, results that also agrees with our previous study that demonstrated that apigenin induces apoptosis in C6 rat glioma cells [14].

An increase in the proportion of GFAP- and β-III-tubulin-positive cells, along with a reduction in nestin expression in cytoplasmic filaments, was observed. This highlights the potential of apigenin to promote the differentiation of tumor cells into cells that exhibit normal and mature astrocyte and neuronal morphology. Although some tumor cells may resist the cytotoxic effects of apigenin, we found that this flavonoid still induced differentiation in the remaining adherent cells after treatment. Immunocytochemical staining analyses of cytoskeletal protein markers at various stages of differentiation and over time revealed that apigenin induced and accelerated morphological changes. Moreover, the expression of markers for mature astrocytes and neurons increased over time, as demonstrated in long-lasting glioblastoma stem cell cultures.

Malignant glioma cells exhibit a strong tendency to infiltrate surrounding brain tissue, which poses a significant challenge for effective therapy [26]. The flavonoid apigenin demonstrated an anti-migratory activity, potentially related to its previously observed antiproliferative and proapoptotic effects in U-251 glioblastoma cells. These findings are supported by other studies reporting similar anti-migratory effects of flavonoids on glioma cells [15,28].

Our in vivo experiments conducted with control, untreated GBM cells have yielded results similar to those reported in the literature regarding tumor formation and progression following the injection of U-251 cells into the rat brain [15,29]. We observed that implanted glioblastomas showed a high cellular density, nuclear pleomorphism, the presence of necrosis, and a large number of multinucleated cells, indicating a high mitotic activity. Similar to our observations, Mercurio et al. [29] and Wang et al. [30], observed reactive astrogliosis in astrocytes adjacent to the tumor in xenotransplants of untreated GBM cells. However, this reaction is not uniform throughout the cerebral hemispheres, indicating a distinct response profile in each brain region. We observed that in the animals that received xenotransplants of GBM cells treated with apigenin, the contralateral hemisphere of the tumor exhibited higher expression of the microglial marker Iba-1 compared to the tumor-implanted region; the proportion of microglia in the areas surrounding tumor cell implants was found to be similar. The immunomodulatory effects of apigenin were also evident in the in vivo tumorigenic assay, where there was a decrease in the expression of mRNA for inflammatory factors such as IL-1β, TNF, and NOS2, along with a downregulation of regulatory factors (IL-10, IL-4) in the brain areas where glioma cells had been implanted after pretreatment with flavonoids that reflect on its growth and microenvironment. Notably, there was a predominance of microglia displaying an amoeboid phenotype, which is characteristic of activated microglia, and these cells were more densely located within the tumor area. NOS2 and its enzymatic product nitric oxide (NO), play a crucial role in the pathophysiology of several inflammatory disorders, and has been suggested as an interesting therapeutic target for malignant tumors, including GBM [31]. Also, it is known that IL4 contributes to the immune resistance seen in GBM regulating apoptosis evasion, promoting self-sufficiency in growth signals and insensitivity to anti-growth signals, besides controlling invasion the replicative potential and sustained angiogenesis [32], and IGF-1 signaling and its cognate receptor IGF-1R is relevant in regulating cell growth and cytokine secretions by GBM and implicated in tumor development and progression and can induce apoptosis following functional inhibition [33]. Moreover, in the GBM tumor microenvironment the cytokine IL-10 is positively regulated, contributing to microglia maintaining a M2-like phenotype, and has a major role in modulating the activity of infiltrating immune cells, predominantly conferring an immunosuppressive action and has been considered as a target to treatment strategies. [9,34]. On the other hand, IL-1 and TNF are pro-inflammatory cytokines that promote the growth, invasion, and immunosuppressive characteristics of GBM [35,36]. Through analysis, we observed a significant reduction in regulatory and pro-inflammatory mRNA levels in the brain implant area of apigenin-treated GBM cells, associated with change in the morphology state of activation of microglia. In a previous study, we also observed that apigenin treatment of microglia/C6 co-cultures resulted in a reduction in C6 cell viability and promoted a microglia-activated phenotype, which was accompanied by an altered TNF/IL-10 ratio, indicating that the flavonoid influences the immune response of microglia towards glioma cells, exhibiting significant antitumor and immunomodulatory properties [22]. Additionally, the ability of the flavonoid to cross the blood-brain barrier suggests considerable biological effectiveness against tumor cells [37], further supporting its potential as a therapeutic agent.

For instance, it is now known that oxidative stress critically influences the pathophysiology of GBM. Recently, it was demonstrated that inhibiting cyclooxygenase-2 (COX-2), which plays a key role in GBM chemoresistance and supports a pro-survival phenotype, in association with TMZ, disrupted redox homeostasis and overcame TMZ resistance [38]. A high number of studies have demonstrated apigenin and antioxidant properties as one of its well-known pharmacological activities and nutraceutical potential [39,40], and antioxidant mechanisms in the GBM context must also be investigated in combination treatments. Importantly the apigenin concentration selected in the study is pharmacologically relevant and potentially translatable to in vivo studies. As revised by Charrière et al. [40] in vivo studies conducted in cancer and cardiovascular diseases models resulted in positive therapeutic effects, also demonstrated in studies of neurodegenerative diseases models. Further pre-clinical and clinical studies with apigenin in the context of GBM will support its use as an adjuvant to the treatment of human glioblastomas.

## 5. Conclusions

In the present study, we showed that apigenin inhibited growth and migration, and induced differentiation, autophagy, and apoptosis in chemoresistant U251 GBM cells. Apigenin anti-glioblastoma effects were also evidenced in GBM stem cells TG1 and OB1, which was a promising result. In addition, it reduced U251 GBM cells’ capacity to form tumors in rat brains, effects associated with the modulation of immunoregulatory proteins, such as IL-1β, TNF, IL-4, and IGF-1. These findings highlight the potential of apigenin as an adjuvant therapy for glioblastoma. Further preclinical and clinical studies are warranted to explore its mechanisms, including its impact on GBM stem cells and oxidative stress pathways, as well as to evaluate its efficacy in combination treatments.

## Data Availability

Data is contained within the article.
